# Changes in Subcortical Structure Volumes Associated With Obesity

**DOI:** 10.7759/cureus.106274

**Published:** 2026-04-01

**Authors:** Sultan Alamri

**Affiliations:** 1 Department of Radiological Sciences, College of Applied Medical Sciences, Taif University, Taif, SAU

**Keywords:** freesurfer, imaging, mri, neuroimaging techniques, obesity

## Abstract

Background

Obesity has been increasingly linked to structural alterations in the brain, particularly involving subcortical gray matter regions. This study aimed to investigate whether obesity is associated with measurable volumetric changes in key subcortical structures relative to normal-weight individuals.

Methodology

In this cross-sectional observational study, 46 healthy volunteers (25.07 ± 5.82 years old) were initially recruited, of whom 38 normal-weight men and eight obese men (body mass index greater than 30 kg/m²) were included. High-resolution T1-weighted MRI scans were acquired and analyzed using both FreeSurfer’s automated segmentation and manual segmentation via ITK-SNAP. Statistical comparisons were performed to determine volumetric differences between groups.

Results

Automated segmentation results revealed significantly larger gray matter volumes in the right amygdala and left thalamus among obese individuals, with near-significant increases observed in the bilateral hippocampus and putamen. Manual segmentation demonstrated significant enlargement of the amygdala and caudate in obese participants.

Conclusions

Collectively, these findings indicate that obesity is associated with subtle but consistent volumetric expansion in several subcortical regions. Further research is needed to clarify the mechanisms underlying these structural differences and their potential cognitive and behavioral implications.

## Introduction

Obesity is characterized clinically by excessive accumulation of body fat and defined by a body mass index (BMI) greater than 30 kg/m². Although it is often perceived as a lifestyle-related condition, obesity is now firmly recognized as a multifactorial medical disorder that substantially increases the risk of numerous health complications, including metabolic, cardiovascular, respiratory, and neurological conditions [1]. It has emerged as a global public health crisis, driven by complex interactions between biological predisposition, behavior, environmental exposures, and socioeconomic factors [2]. By the turn of the century, obesity had reached epidemic proportions worldwide, becoming one of the leading contributors to mortality and disability [3]. Numerous determinants, such as family history [4], the fat-mass and obesity-associated (*FTO*) gene [5], diabetes, cardiovascular disease, sleep apnea, and various malignancies, have been implicated in the clinical burden of obesity [6].

In Saudi Arabia, obesity presents a particularly significant challenge. Recent national data indicate that the country ranks 14th globally in adult obesity prevalence, with 35.4% of adults classified as obese in 2022 and a national weighted prevalence of 24.7% reported in 2020 [7]. Sedentary lifestyles, declining physical activity among younger populations, and widespread adoption of energy-dense dietary patterns have been proposed as major contributing factors [8].

BMI remains the most used metric to categorize weight status based on height and age. According to the World Health Organization (WHO), individuals are classified as underweight (<18.5 kg/m²), normal weight (18.5-25 kg/m²), overweight (26-30 kg/m²), and obese (>30 kg/m²) [9,10]. However, increasing evidence suggests that central adiposity measures such as waist-to-hip ratio (WHR) may be more informative predictors of obesity-related brain changes [11]. WHR provides an index of visceral fat distribution, which has been strongly associated with neuroanatomical alterations and brain atrophy [12]. For example, greater WHR has been linked to reduced hippocampal volume (p = 0.02) and other adverse neurological outcomes [13,14].

Beyond metabolic disease, obesity has been connected to alterations in brain structure and function. Research has demonstrated associations between obesity and reduced gray matter volume, impaired white matter microstructure, and neuroinflammatory processes [11,15]. Obesity has also emerged as an important risk factor for cognitive decline and Alzheimer’s disease in older adults [16,17]. In younger individuals, it has been associated with reduced memory performance, altered executive function, and accelerated brain aging [18]. Obesity is additionally linked to decreased cerebral blood flow in prefrontal regions involved in attention, reasoning, and behavioral regulation [11].

Growing evidence highlights structural gray matter abnormalities across multiple cortical and subcortical regions in obese individuals [19]. Studies have reported reduced gray matter volumes in the inferior frontal gyri, insula, precentral gyri, temporal cortex, amygdala, and cerebellum [20-30], while other regions, including the cuneus and occipital areas, may show relative enlargement. Subcortical structures also appear particularly sensitive to obesity-related changes. Increased volumes of the thalamus, putamen, and globus pallidus have been reported, along with reduced volumes in the caudate nucleus [20]. Even overweight individuals (BMI = 25-30 kg/m²) exhibit early signs of basal ganglia atrophy and compromised white matter integrity [31].

Subcortical regions are deeply implicated in feeding behavior, motivational processing, and reward regulation. A recent study by Gómez-Apo et al. (2021) demonstrated associations between obesity and structural variations in subcortical nuclei involved in sensory integration and appetite regulation [20]. The thalamus, hippocampus, and basal ganglia, including the caudate and putamen, are fundamental components of neural circuits governing satiety, reward processing, memory, and learned associations related to food [32,33]. The amygdala, in particular, plays a pivotal role in emotional and appetitive responses, integrating sensory input related to food cues and contributing to eating behaviors [34,35]. The caudate participates in reward-based learning and cognitive control over eating, while dysfunction in its circuitry has been linked to overeating and weight gain [36].

Despite the expanding literature, the precise pattern and direction of obesity-related changes in subcortical structures remain inconsistent across studies. Differences in methodology, sample characteristics, and imaging protocols may contribute to these discrepancies. Therefore, the present study aims to examine structural variations in five key subcortical regions, namely, the amygdala, thalamus, putamen, hippocampus, and caudate, among obese and non-obese men. By using both automated and manual segmentation methods, this study seeks to provide a clearer understanding of how obesity affects subcortical anatomy in young adult males.

## Materials and methods

This cross-sectional observational study was approved by the Ministry of Health, Directorate of Health Affairs, Taif governorate, and written informed consent was obtained from the Department of Research and Studies (IRB registration number with KACST, KSA: HAP-02-T-067, approval number 502 on 12/30/2020).

Participants

The data was collected from King Faisal Medical Complex in Taif City from 46 healthy volunteers (25.07 ± 5.82 years old). All volunteers were scanned using the Siemens 3-Tesla MRI scanner. Volunteers were chosen based on an online safety checklist questionnaire to ensure their eligibility to be included in this study. Each was coded numerically for confidentiality. The exclusion criteria for participation in this research project were defined to ensure participant safety and maintain the integrity and quality of the imaging data. Individuals were excluded at two stages, i.e., during initial screening and on the day of imaging. During the initial screening phase, volunteers were excluded if they reported a history of significant medical conditions that could affect study outcomes or participant safety. Specifically, individuals with a history of cardiac surgery were excluded, as well as those who reported experiencing migraines. On the day of imaging, additional exclusion criteria were applied based on factors that could interfere with the successful completion or quality of the MRI scans. Participants who experienced claustrophobia were excluded due to their inability to tolerate the scanning environment. Furthermore, individuals with orthodontic braces were excluded because metallic components can produce susceptibility artefacts, leading to signal distortion and anterior signal loss in the acquired images. These criteria were implemented to ensure both the safety of participants and the acquisition of high-quality, reliable imaging data suitable for analysis. The remaining volunteers (n = 38) met the study criteria and had no contraindication for undergoing an MRI scan (e.g., pacemakers, aneurysm clips, metal stents, artificial implants). In addition, data from eight obese adults with a BMI greater than 30 kg/m² were obtained for this research.

Imaging protocol

All volunteers were scanned on an MRI scanner (Siemens MAGNETOM Skyra 3 T). The imaging protocols (Table 1) were optimized based on the proposed protocol by the developers of FreeSurfer (an open-source software package available for the analysis and processing of anatomical brain neuroimaging data).

**Table 1:** MRI brain imaging protocol.

Items	Siemens Scanner (Parameters)
Protocol	Magnetization Prepared RApid Gradient Echo (MPRAGE)
Voxel size (mm³)	1.0 × 1.0 × 1.0
Field of view (mm)	256 (256 × 256 matrix)
Slices orientation	176 sagittal slices (3D)
Slice thickness (mm)	1
Repetition time (ms)	2,530
Inversion time (ms)	1,100
Echo time (ms)	1.64

Image analysis

All images were visually inspected at the time of data acquisition to ensure there were no severe motion artifacts or any other artifacts. All Digital Imaging and Communications in Medicine (DICOM) images were converted to Neuroimaging Informatics Technology Initiative (NIFITI) format using Medical Image Processing, Analysis, and Visualization (MIPAV; version 9.0.0) software. The images were then analyzed using FreeSurfer’s fully automated command without any interventions, “recon-all-i input_image.nii -all” (version 7.1.1, https://surfer.nmr.mgh.harvard.edu/), by applying the longitudinal stream [37]. The software version, computing hardware, and operating system (Ubuntu 20.04.2 LTS) were consistent for this study, as recommended by Gronenschild et al. (2012) [38], to avoid changes related to different processing conditions. The FreeSurfer results were also visually inspected for any misregistration. The images were also manually analyzed using ITK-SNAP software (version 3.8.0) by two observers. Regions of interest in T1-weighted imaging MRI were segmented, and then the volumes of the segmented structures were generated to be used for statistical analysis [39].

Statistical analysis

We used SPSS (IBM Corp., Armonk, NY, USA) to identify if there was any obesity-caused effect on the size of the subcortical structures. We performed an independent-samples t-test to check for any significant change between the two variables (obese and control) on FreeSurfer (automated segmentation) and ITK-SNAP (manual segmentation). At p-values (significance, two-tailed) of less than or equal to 0.05, we could conclude that the mean difference between our variables was statistically significant.

## Results

Using FreeSurfer’s automated segmentation, we first compared volumetric measurements of each subcortical structure between obese and normal-weight participants (Table 2). Across all structures examined, obese individuals demonstrated larger average gray matter volumes. Statistically significant increases were observed in the right amygdala (p = 0.011) and left thalamus (p = 0.032). Although several additional regions did not meet the threshold for statistical significance, they exhibited notable trends toward enlargement in obesity. These included the right putamen (p = 0.053), left putamen (p = 0.072), right hippocampus (p = 0.086), and left hippocampus (p = 0.087). No significant differences were found between groups in the right thalamus, left amygdala, or bilateral caudate volumes.

**Table 2:** Comparison of volumetric measurements of various subcortical structures using FreeSurfer.

Structure	Mean (obese)	Mean (control)	t	P-value
Right amygdala	1,944.7	1,740.0	2.426	0.011
Left amygdala	1,815.1	1,713.2	1.204	0.235
Right thalamus	8,258.1	7,900.3	1.034	0.307
Left thalamus	8,629.1	7,933.1	2.215	0.032
Right hippocampus	4,472.9	4,258.7	1.753	0.086
Left hippocampus	4,386.2	4,168.9	1.752	0.087
Right putamen	6,019.2	5,586.7	1.989	0.053
Left putamen	5,926.1	5,534.6	1.841	0.072
Right caudate	3,947.9	3,762.3	1.000	0.323
Left caudate	5,175.4	4,948.0	0.798	0.429

To further validate the automated results, manual segmentation was performed using ITK-SNAP for each subcortical region (Table 3). Manual measurements revealed enlargement in the amygdala and caudate among obese individuals. Importantly, statistical significance was detected for both the right and left amygdala, as well as the right and left caudate (p < 0.05). These findings are consistent with the automated analysis in showing that the amygdala demonstrates the most robust volumetric increases associated with obesity, while the caudate, despite showing non-significant trends in the automated analysis, emerged as significantly enlarged when measured manually.

**Table 3:** Comparison of volumetric measurements of various subcortical structures using ITK-SNAP.

Structure	Mean (obese)	Mean (control)	t	P-value
Right amygdala	2,264.5	1,833.4	3.097	0.003
Left amygdala	2,244.9	1,732.4	3.950	0.0002
Right thalamus	7,367.6	7,408.7	-0.180	0.858
Left thalamus	7,143.9	7,231.6	-0.372	0.712
Right hippocampus	3,697.9	3,648.8	0.307	0.76
Left hippocampus	3,595.9	3,48.7	-0.773	0.443
Right putamen	5,433.8	5,535.0	-0.494	0.624
Left putamen	5,515.0	5,559.2	-0.211	0.834
Right caudate	5,221.8	4,495.8	2.884	0.006
Left caudate	5,061.7	4,394.9	2.833	0.007

Together, these results indicate that obesity is associated with measurable expansion in several subcortical structures, with the most consistent effects observed in the amygdala and caudate, and additional trends suggesting enlargement in the thalamus, putamen, and hippocampus.

## Discussion

This study explored volumetric differences in key subcortical structures between obese and non-obese men using both automated and manual MRI segmentation methods. The overall findings indicate that obesity is associated with increased gray matter volume across all examined regions, with the amygdala and thalamus showing the most prominent and statistically significant changes in the automated analysis, and the amygdala and caudate showing significant enlargement with manual segmentation.

The amygdala, a structure central to emotional regulation and appetite-driven behaviors, demonstrated particularly strong associations with obesity. Our findings support earlier work by Widya et al. (2011) and Perlaki et al. (2018), who similarly observed larger amygdala volumes in individuals with higher BMI [40,41]. The lateralization pattern observed, i.e., significant enlargement in the right amygdala and a non-significant increase on the left, mirrors evidence suggesting functional and structural asymmetry. Prior studies have documented hemisphere-specific amygdala functions, such as the right amygdala’s role in fear conditioning [42,43] and its involvement in affective responses, including ictal orgasmic auras [44]. Several mechanisms may underlie amygdalar enlargement in obesity, including elevated glucose metabolism, altered cerebral blood flow, and potential increases in glial cell populations or interstitial fluid [45,46]. Because amygdala enlargement is also seen in other conditions such as depression, psychosis, autism, and bipolar disorder [46-49], its structural plasticity may reflect complex interactions between metabolic, inflammatory, and emotional processes associated with obesity.

The thalamus also showed a significant volumetric increase among obese participants in the automated analysis. While previous studies have reported enlarged thalamic volumes in obesity [20,50-53], the underlying mechanisms remain poorly understood. It is plausible that thalamic enlargement may relate to genetic factors such as the *FTO* gene [5], developmental influences, or alterations in sensory-processing circuits related to feeding behavior. Younger age has also been associated with greater thalamic volume [20], which may partially explain the trend observed in this relatively young cohort.

Although the hippocampus did not show statistically significant differences, both automated and manual analyses indicated modest volumetric increases in obese individuals. Prior research has reported mixed results, with some studies showing reduced hippocampal volume in obesity while others demonstrate hypertrophic changes [40,53]. The hippocampus plays a critical role in linking memory, context, and learned associations to feeding behavior, including episodic meal memories and conditioned responses to food-related cues [32]. Structural enlargement may therefore reflect adaptive or maladaptive changes in memory-based regulation of eating.

The putamen also demonstrated near-significant enlargement in obese participants. This is consistent with findings by Gómez-Apo et al. (2021) and others, who have linked BMI to increased putamen volume and altered striatal activity [20,54]. The putamen participates in reward processing and insulin signaling regulation, mechanisms thought to contribute to impaired appetite control in obesity. Dysregulated putaminal structure and function may therefore represent a neural correlate of heightened food motivation and diminished inhibitory control.

Contrary to several previous studies reporting reduced caudate volume in obesity [20,31,55], our manual segmentation results showed that both caudate nuclei were significantly enlarged in obese individuals. The reasons for this discrepancy are unclear but may reflect methodological differences, the small sample size, population characteristics, or early-stage neuroplastic adaptations to overeating. The caudate is deeply involved in reward processing, decision-making, and cognitive control over food intake [36]. Reduced caudate responses to palatable food consumption have been associated with weight gain [56,57], suggesting that obesity may involve complex structural and functional modifications over time.

This study is subject to several limitations. The relatively small sample size, particularly within the obese group, may limit the statistical power and generalizability of the findings. Furthermore, the inclusion of only young adult males restricts the applicability of the results to other populations. Obesity classification was based solely on BMI, without the incorporation of more precise measures of adiposity or fat distribution. In addition, potential confounding variables, including lifestyle and metabolic factors, were not controlled for in the analysis. Methodologically, both automated segmentation using FreeSurfer and manual segmentation using ITK-SNAP are associated with inherent limitations.

## Conclusions

Based on both automated and manual segmentation methods, this study demonstrates that obesity is associated with volumetric enlargement across multiple subcortical structures in young adult men. FreeSurfer-based automated analysis revealed significant increases in the right amygdala and left thalamus, while manual segmentation identified significant enlargement in both amygdalae and both caudate nuclei. Although some structures showed non-significant trends, the overall pattern supports the notion that obesity contributes to subtle but widespread alterations in subcortical anatomy. These findings highlight a potential association between obesity and structural alterations in subcortical brain regions. In particular, the observed volumetric differences may suggest effects on neural circuits implicated in reward processing, emotional regulation, feeding behavior, and metabolic balance. However, because this study focused solely on subcortical volume changes and did not directly assess functional or circuit-level abnormalities, these interpretations should be considered hypotheses rather than definitive conclusions. Future studies integrating functional imaging and pathway analyses are warranted to directly evaluate the impact of obesity on neural circuits underlying these processes. Future research employing larger sample sizes, longitudinal designs, and additional measures such as WHR is warranted to clarify the mechanisms driving these structural differences and their potential functional consequences.
